# How Bats Land Upside Down

**DOI:** 10.1371/journal.pbio.1002298

**Published:** 2015-11-16

**Authors:** Robin Meadows

**Affiliations:** Freelance Science Writer, Fairfield, California, United States of America

## Abstract

A new study reveals that bats use the inertia of their unusually heavy wings—rather than their aerodynamic properties—to help them perform acrobatic maneuvers like landing upside down to roost. Read the Research Article.

Bats are nimble aerialists, neatly flipping over to roost in caves, hollow trees and other sheltered spots. But the mechanics of this feat have been a mystery, as bats cannot hover upside down. How, then, do they manage to land head-under-heels without falling toward the ground? In this issue of *PLOS Biology*, Breuer and colleagues provide an answer as elegant as the acrobatic maneuvers of bats: to flip their bodies while landing, bats simply retract one wing and extend the other.

Why is this all it takes to turn a bat upside down? Bats take advantage of the fact that their wings are heavy with bone and muscle. Think of a figure skater who spins faster by pulling her outstretched arms to her chest—by moving the mass of her arms closer to her body, she decreases her moment of inertia, which in turn increases her spinning speed. Similarly, a bat can roll over swiftly by moving the mass of its wings relative to its body. Called inertial reorientation, this mode of movement also explains why cats land on their feet when they fall upside down. It also underpins the aerial agility of lizards and rodents, and of people while somersaulting and diving.

To test whether bats use wing inertia to land upside down, the researchers took high-speed videos of two species—Seba's short-tailed bats (*Carollia perspicillata*) and Lesser dog-faced fruit bats (*Cynopterus brachyotis*)—as they landed, or attempted to land, on a ceiling. Tracking specific points on bat bodies and wings let the researchers analyze the complex movements of bats during this maneuver (Fig 1). A close look at a Seba's short-tailed bat revealed that during the transition from flight to landing upside down, wing movements change dramatically. As the bat neared the ceiling, it slowed the flapping of its wings. Then, in quick succession, it retracted its wings on an upstroke, extended them on the next downstroke and—at the moment of flipping over—pulled one wing towards its body. As the bat flipped, the retracted wing stayed near the ceiling while the head and extended wing hung toward the floor. At the same time, the bat reached for the ceiling with its feet. All this took only half a second.

**Fig 1:**
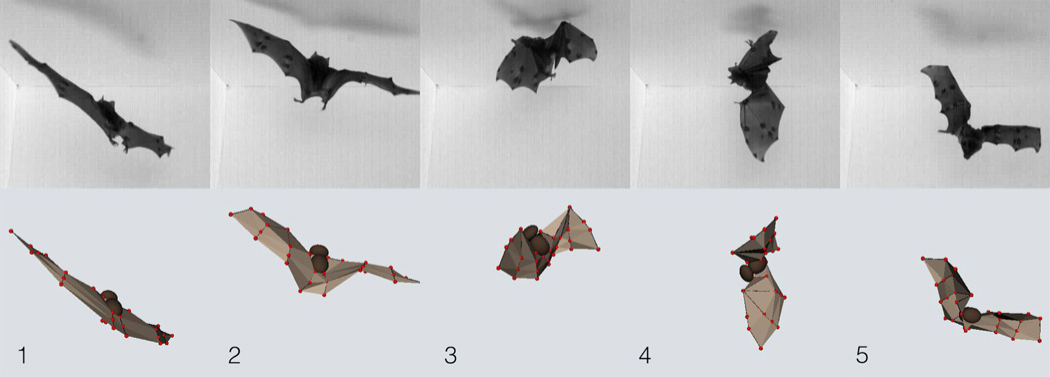
Tracking and modeling a bat landing attempt. A Seba’s short-tailed bat attempts to make a ceiling landing, but finding its landing site blocked, begins to fall, re-orients, and regains a stable flight trajectory, continually repositioning and reconfiguring its wings to help control body orientation and trajectory. Selected frames from high-speed videography (one camera of three employed) shown in top row; 3-D computational model of the same bat, reconstructed from the video images, shown in bottom row. *Image credit*: *Attila Bergou*.

Using two models of bat flight, the researchers confirmed that a shift in wing inertia is enough to flip a bat. The first is a simple model that limits wings to flapping up or down, and extending or retracting. This model shows that when one wing retracts, the bat does indeed roll upside down. Moreover, the result is identical when the aerodynamic forces that power flight are set to zero, strengthening the conclusion that wing inertia can flip a bat. Furthermore, heavy wings are a must for inertial reorientation: the model predicts that a fruit fly, which has nearly massless wings compared to its body, relies solely on aerodynamic forces to execute this maneuver.

The second model mimics all the complex movements of bats—including rapid, precise changes in body and wing position as well as in the angles of the many joints in each wing—as they rotate in the air to land upside down or to right themselves after a botched landing. Again, simulations show that wing inertia is key to reorienting airborne bats. The same would hold for bats as they drop from their roosts and turn over to fly.

As all bats have massive wings and most species roost upside down, these findings likely apply broadly across bats. In addition, the use of inertial maneuvering by many vertebrate lineages suggests that it evolved either early or multiple times. The researchers also propose that by providing control during leaping and gliding, inertial maneuvering may have been a step along evolutionary pathways to powered flapping flight. And by finding that wing inertia confers maneuverability at slow speeds, this research also shows that heavy wings are not always a liability to flight. This counterintuitive conclusion may further development of winged robots inspired by bats, insects, and birds.
